# Optoacoustic Imaging Offers New Insights into In Vivo Human Skin Vascular Physiology

**DOI:** 10.3390/life12101628

**Published:** 2022-10-18

**Authors:** Luis Monteiro Rodrigues, Tiago F. Granja, Sergio Faloni de Andrade

**Affiliations:** CBIOS-Research Centre for Biosciences and Health Technologies, Universidade Lusófona Av, Campo Grande 376, 1749-024 Lisboa, Portugal

**Keywords:** optoacoustic tomography, PAT, vascular physiology, reactive hyperemia, human upper arm

## Abstract

Functional imaging with new photoacoustic tomography (PAT) offers improved spatial and temporal resolution quality in in vivo human skin vascular assessments. In the present study, we followed a suprasystolic reactive hyperemia (RH) maneuver with a multi-spectral optoacoustic tomography (MSOT) system. A convenience sample of ten participants, both sexes, mean age of 35.8 ± 13.3 years old, was selected. All procedures were in accordance with the principles of good clinical practice and approved by the institutional ethics committee. Images were obtained at baseline (resting), during occlusion, and immediately after pressure release. Observations of the RH by PAT identified superficial and deeper vascular structures parallel to the skin surface as part of the human skin vascular plexus. Furthermore, PAT revealed that the suprasystolic occlusion impacts both plexus differently, practically obliterating the superficial smaller vessels and evoking stasis at the deeper, larger structures in real-time (live) conditions. This dual effect of RH on the skin plexus has not been explored and is not considered in clinical settings. Thus, RH seems to represent much more than the local microvascular reperfusion as typically described, and PAT offers a vast potential for vascular clinical and preclinical research.

## 1. Introduction

Vascular functional imaging has generated a considerable amount of interest in the last years in preclinical and clinical research. Knowledge of vascular physiology and pathophysiology is critical to the understanding of disease mechanisms [1,2,3,4], and recent developments in non-invasive combined sound- and light-based technologies have provided new directions of exploration [1,3,4,5]. Photoacoustic tomography (PAT) depicts acoustic waves generated by the thermoelastic expansion of structures, typically endogenous chromophores such as hemoglobin and melanin, due to the absorption of light [3,5,6,7,8]. This expansion can be quantified, providing real-time information on the circulatory status of the assessed body region.

PAT is regarded as a deep-tissue imaging technology, not comparable to surface imaging techniques such as capillaroscopy and confocal microscopy or to other intermediate imaging techniques such as OCT (optical coherence tomography) [3,8,9]. It is accepted that PAT penetrates deeper with high-resolution than optical tomography and offers better contrast with fewer artifacts than high-resolution sonography [3,5,6,10]. In fact, using ultrasound in place of optical detection eliminates photon scattering. Moreover, considering other comparable technologies, PAT is fast, accurate, and does not use ionizing radiation. For all these reasons, the development of PAT applications has increased in various domains, although with a particular interest in cardiovascular biology. Human skin is often targeted as a primary organ due to its accessibility and the real-time structural, molecular, and metabolic information which may be provided by PAT [3,10,11,12,13,14].

Our research follows these views, assuming that PAT provides a different analytical perspective and a deeper look into these multiple complexities of vascular physiology and pathophysiology [1,2,5,15]. With the (specific) thermoelastic expansion measured, PAT enables a vessel-by-vessel quantification of perfusion-related chromophores, supplying functional information in different (spatial) planes that may allow researchers and physicians to map each circulatory region. By extension, multiple wavelengths of light in multi-spectral optoacoustic tomography (MSOT) enable the separation of different chromophores, reportedly with higher spatial resolution and penetration capacities, and wavelength unmixing and detailed image reconstruction is made with powerful post-acquisition processing [1,2,3,4,5,16].

In the present study, we use an MSOT system to follow the live impact of reactive hyperemia (RH), a classical experimental strategy used to challenge cardiovascular adaptive capacity. Although long used as a predictor of cardiovascular impairment, many questions remain regarding the mechanisms, significance, and applicability of RH responses [17,18,19,20]. Here we explore the applicability of the MSOT technology in healthy human vascular physiology by assessing the RH evoked in the flexor forearm by the suprasystolic occlusion of the brachial artery. By the diversity of information provided, we also aim to better understand the adaptive intervenients in RH of the upper limb.

## 2. Materials and Methods

### 2.1. MSOT

The MSOT optoacoustic system from iThera Medical GmbH (Munich, Germany) was used. The technical specifications and principles of the system have been detailed elsewhere [5,6,8,16,21,22,23]. In brief, PAT detects sound waves generated when molecules absorb optical light from a wave laser beam. This evokes a transitory thermoelastic expansion that allows the identification of specific (excited) chromophores, as the signal pattern across the wavelengths measured serves as an exclusive absorption feature for each (chromophore) molecule [5,6,7].

### 2.2. Study Population

This exploratory study included ten healthy participants of both sexes, 18 to 60 years old, selected after specific inclusion/non-inclusion criteria and previously informed about the study’s purposes and procedures. The primary selection aspects were normotensive, nonsmokers, and free of any chronic disease demanding regular medication or food supplementation. The body mass index (BMI) and mean arterial pressure (MAP) were calculated for all participants (Table 1). Caffeine and alcohol consumption was restricted during the 24 h prior to measurements, as was the application of any topical (including cosmetic) products to the surface of the skin. The study followed the principles of good clinical practice established for human research [24] and was previously approved by the institutional Ethics Committee (Process CE.ECTS/P10.21).

### 2.3. Experimental

Before measurements, volunteers were allowed to adapt to the laboratory temperature, humidity, and light (20–30 min).

Circulatory functional imaging was obtained under dynamic conditions through a post-occlusive reactive hyperemia maneuver performed in the upper limb. The pressure cuff was applied in the middle arm while measurements were obtained in the ventral forearm, with the MSOT measurement probe, a 3D cup fixed to the surface of the forearm by a flexible metal arm [16]. After baseline scan acquisition, the cuff was rapidly inflated with 200 mmHg to occlude the brachial artery. This pressure was maintained for one minute to ensure hemodynamical stabilization in the area. The cuff was then rapidly deflated, and videos were recorded during the immediate post-occlusion recovery. Throughout the experimental procedure, the main chromophores oxyhemoglobin HbO_2_ and deoxyhemoglobin Hb were simultaneously visualized (in real-time) by the MSOT system.

Videos were post-processed for image reconstruction by the ViewMSOT software (iThera Medical version 4.0). MSOT software was used to collect data from selected regions of interest (ROI) and to quantify HbO_2_, Hb, HbT (total hemoglobin, the sum of HbO_2_ and Hb), and the mean saturation of oxygen (mSO_2)_ during the different stages of the experiments. Image J software (National Institutes of Health, version 1.53k14) was also used in the image reconstruction process.

### 2.4. Statistical Analysis

Statistical analysis was performed in GraphPad Prism 9.2.0.283 MachineID: 0861F12DB8D10. Normal distribution was tested with the Kolmogorov–Smirnov test and direct observation of generated QQ plots.

## 3. Results and Discussion

**Figure 1:**
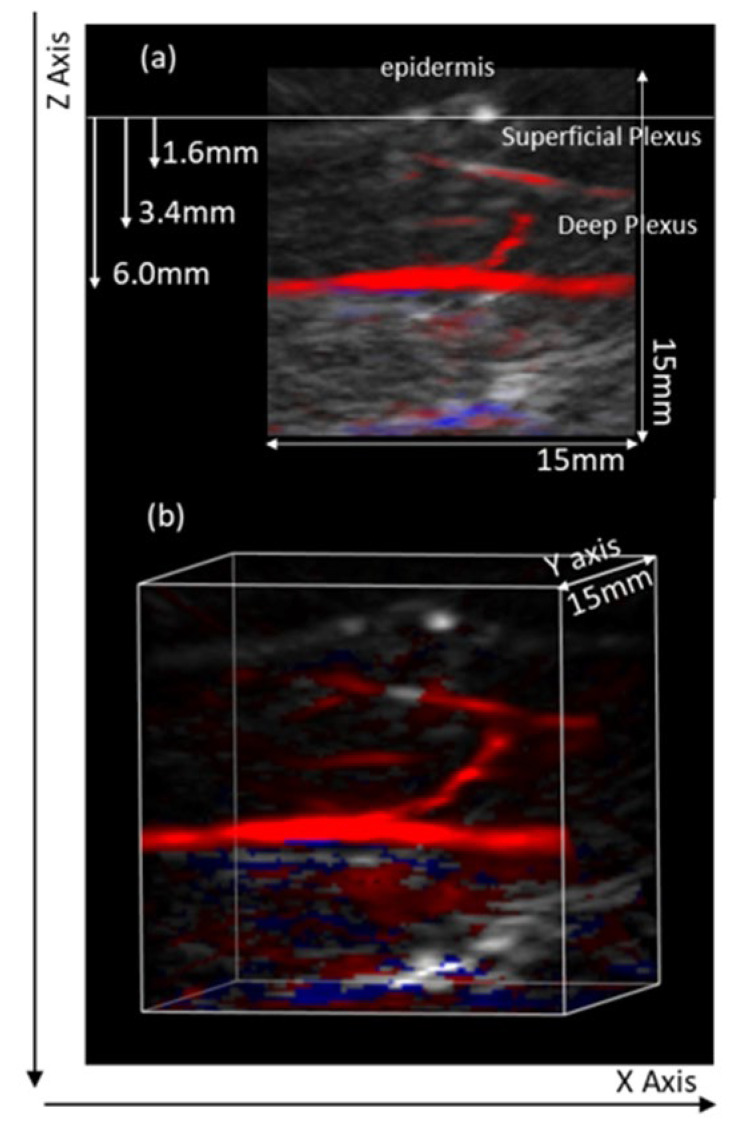
Illustrative images from the flexor aspect of the human forearm obtained with the MSOT technology. (**a**) Different vascular structures parallel to the skin surface are seen at different depths and assumed to correspond to the superficial and the deep skin plexuses; the different diameters seen are consistent with this assumption. The vascular structure running perpendicular likely corresponds to an arteriole connecting both plexuses. Some deeper structures might also be present in the same ROI. (**b**) The same structures shown in a 3D view.

MSOT scans provided images in frontal, transversal, and longitudinal planes, from which various ROIs might be selected for further calculations. Baseline scans obtained in the (central) flexor forearm were compared with the occlusion and post-occlusion scans in the same area. High-resolution images were assessed at different depths detailing the skin microcirculation organization at this site. PAT consistently revealed two vascular plexuses parallel to the skin surface—larger vessels located 2 to 6 mm below the skin surface corresponding to the deep plexus and smaller vessels located 0.6 to 2 mm below the skin. The capillary structures corresponding to the papillary dermis were difficult to visualize (Figure 1). Perpendicular structures connecting both plexus and deeper vascular structures, likely crossing subcutaneous tissue and/or muscle, were also visible (Figure 1). Considering this resolution capacity, the aim of our study was to visualize and quantify the impact of a real-time RH procedure in both plexuses.

Through the ROI analysis of the MSOT scan images, HbO_2,_ Hb, HbT, and mSO_2_ can be determined [16,21,22]. HbT and SO_2_ are semi-quantitative physiological parameters automatically calculated for each pixel whenever both Hb and HbO_2_ components are unmixed. According to the instrument manufacturer, different fluence and spectral coloring will consistently impair the comparison of these MSOT descriptors with similar from other technologies. Thus, ‘m’ is used to designate MSOT-derived oxygen saturation [22]. HbT refers to the semi-quantitative sum of unmixed deoxy- and oxy-hemoglobin components calculated from all pixels after setting negative pixels in Hb and HbO_2_ to 0 [22]. mSO_2_ represents the MSOT-derived fraction of oxygenated hemoglobin divided by total hemoglobin, where mSO_2_ is calculated if (and only if) a pixel has Hb > 0 and HbO_2_ > 0 [22].

**Figure 2:**
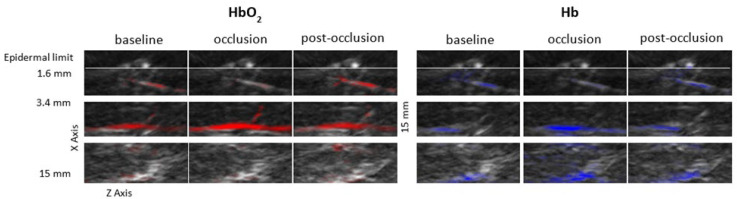
Illustrative images of HbO_2_ (**red**) and Hb (**blue**) from the flexor aspect of the human forearm obtained with the MSOT technology during the reactive hyperemia induced by arterial occlusion with suprasystolic pressure. Different vascular structures parallel to the skin surface are depicted by the different colored chromophores at different depths during the different phases of the experimental protocol and are assumed to correspond to the superficial and the deep skin plexus. Additional subcutaneous structures can also be detected at deeper locations, although with reduced resolution.

**Figure 3:**
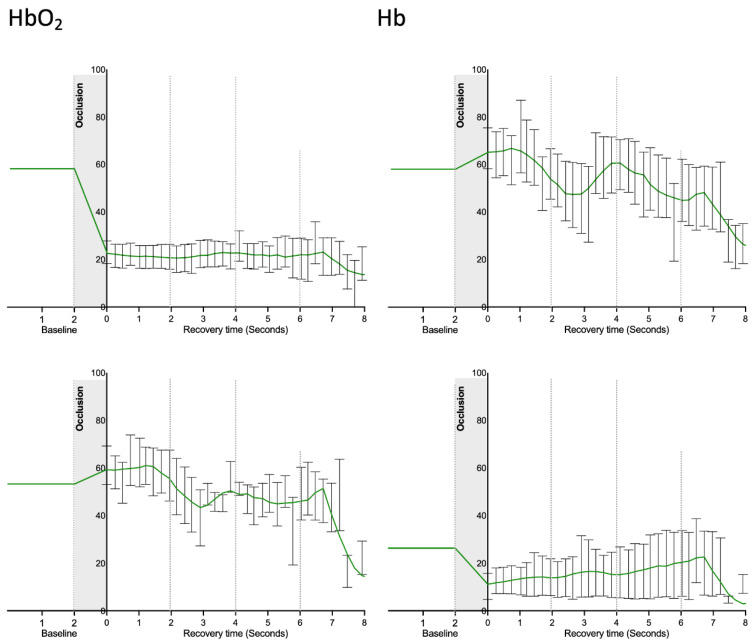
HbO_2_ and Hb signals obtained in the superficial (**top**) and the deeper skin plexus (**bottom**) of healthy participants (n = 10) during the experimental RH protocol. As shown, these parameters progress in different directions during the procedure, indicating that the suprasystolic occlusion has different effects at different depths, affecting global hemodynamics (please see text). Signals are quantified by MSOT based on the identification of the respective chromophores (values correspond to mean and SEM; data distribution was tested with the Kolmogorov–Smirnov test.

**Figure 4:**
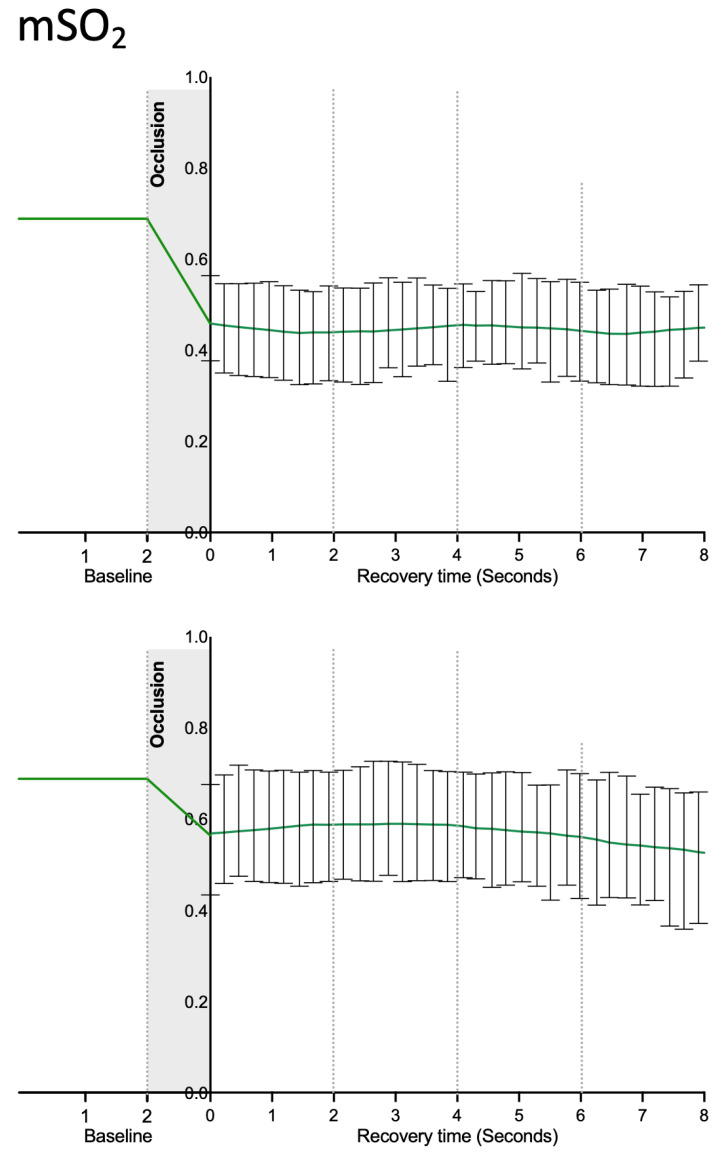
MSOT-derived oxygen saturation (mSO_2_) signals obtained in the superficial (**top**) and the deeper skin plexus (**bottom**) of healthy participants (n = 10) during the experimental RH protocol (please see text). Signals are quantified by MSOT based on the identification of the respective chromophores (values correspond to mean and SEM; data distribution was tested with the Kolmogorov–Smirnov test).

Image reconstruction focused on our chromophores of interest (Figure 2) indicates that the suprasystolic pressure clearly decreases HbO_2_ in the superficial plexus due to the collapse of those vessels, while HbO_2_ seems to increase at the deeper skin plexus, likely due to some transfer of blood from higher structures but also due to stasis and reduction of the capacity of (O_2_) transfer to the tissues. Following the pressure release, the HbO_2_ signal slowly increased at the surface and decreased in the lower skin plexus. During the experiments, Hb concentration profiles consistently evolved in the opposite direction in both plexus (Figure 3). The calculated mSO_2_ decreased in both plexuses with occlusion, with slow recovery (increase) after cuff deflation (Figure 4).

RH is a widely used functional challenger used in vascular research. Mechanisms of this response to ischemia in the human skin microcirculation are not clearly understood. Still, RH is accepted to represent the magnitude of reperfusion in that anatomical location following a (short) period of arterial occlusion [17,18,19,20]. Both metabolic and myogenic components seem to be present, but past studies suggest that sensory nerves play a central role [3,17,25].

A principal concern involves the distinction between venous and arterial occlusion. Measurements are normally performed after occluding macrocirculation vessels (the brachial artery in the arm, as an example) to assess microcirculation through the skin. It is known that venous and arterial systems interact permanently to adjust local and systemic hemodynamics. Therefore, the perfusion changes caused by RH are certainly influenced by venous dynamics during the occlusion–disocclusion process [26,27]. Even so, weak RH reperfusion is commonly regarded as a sign of (micro)vascular impairment [17,19,20].

These questions, partially explained by time-related technological limitations, motivated RH exploration with other instruments in addition to plethysmography and laser Doppler flowmetry. RH has been investigated with near-infrared spectroscopy (NIRS) [28,29] and compared with PAT [30], assessed with diffuse correlation spectroscopy (DCS) [31], contrast-enhanced ultrasound (CEUS) [32], and peripheral artery tonometry [33]. Although these studies confirm the possibility of the use of quantifiable metabolic and molecular variables, together with image(s), in clinical and preclinical settings, there is still no agreement about outcomes. Stronger correlations for the “time-to-peak” variable from CEUS and NIRS were recently reported [32]. Moreover, the venous and arterial involvement in RH is still not properly controlled in typical procedures, meaning that the vascular responsiveness of both micro and macrocirculation at different depths cannot be clearly distinguished [17,28,29,34,35]. Additionally, recent data from DCS suggests that skin circulation is not the best model to study skeletal muscle vasculature by RH [31].

In our study, we demonstrate for the first time, to the best of our knowledge, the in vivo functioning of skin plexus under suprasystolic pressure. While the smaller vessels of the superficial plexus immediately collapse when pressure rises and perfusion is nearly reduced to zero, the opposite effect is observed at the deep plexus, where larger vessels and higher amounts of blood are present with blood accumulating on site, enlarging these structures and increasing their visibility (Figure 2). The main chromophores HbO_2_ and Hb, along with calculated mSO_2_, progress accordingly (Figure 3 and Figure 4). We believe from the present data that the deeper vascular structures shown correspond to the deeper skin plexus and not to the muscle beneath.

It is also clear that the intensity and duration of the occlusion clearly affect the procedure. The separate control of venous and arterial occlusion by using different cuff pressures offers multiple controversies [27,28,30,36]. We opted for the application of 200 mmHg suprasystolic pressure in our study to ensure arterial occlusion, confident that pressure affects multiple structures in depth and involves more than the microcirculatory units in the area. This is in line with previous findings showing that the RH response to the arterial occlusion of one limb was detectable in the contra-lateral non-occluded limb [32,37,38]. This observation suggests that RH involves a centrally mediated control we previously termed as a prompt adaptive hemodynamical response (PAHR) [39].

In the present study conditions, the use of PAT to monitor RH not only fills the gap between morphology and function but also suggests that RH represents much more than the previously believed local microvascular reperfusion. Further, the microcirculatory detail provided with these results indicates that the predictive value of RH in clinical practice still faces important challenges.

Our study should be regarded as exploratory, as our focus was limited and our population small. Nevertheless, it is clear that PAT technologies have great advancement potential for functional vascular imaging, with remarkable clinical potential.

## Figures and Tables

**Table 1:** General characteristics of selected participants (N = 10).

Participants	Mean ± sd
Sex (F female; M male)	F (5); M (5)
Smokers	0
Physical Activity (h/week)	3.0 ± 1.9
Age, years	35.8 ± 13.3
Body mass, kg	68.2 ± 9.8
Height, m	1.7 ± 0.1
BMI, kg/m^2^	23.7 ± 2.5
MAP, mmHg	91.4 ± 4.1

Data tested for normality with D’Agostino and Pearson test in GraphPad Prism with the multi-variable module (Prism version 9 for MacOS); sd—standard deviation.
